# Correlation between Serum 25-Hydroxyvitamin D Levels and Gastric Cancer: A Systematic Review and Meta-Analysis

**DOI:** 10.3390/curroncol29110661

**Published:** 2022-11-02

**Authors:** Xing Liu, Yueyue Zhou, Xihuan Zou

**Affiliations:** School of Public Health, Yangzhou University, No.136 Jiangyang Middle Road, Yangzhou 225000, China

**Keywords:** gastric cancer, diagnosis, serum 25-hydroxyvitamin D, vitamin D

## Abstract

The purpose of this meta-analysis was to evaluate the relationship between serum 25-hydroxyvitamin D[25(OH)D] levels and gastric cancer. PubMed, Embase, Cochrane Library, Web of Science, The China Academic Journals full-text database, Wanfang Database of Chinese Academic Journals, VIP Chinese Science and Technology Periodicals database, and Chinese Biomedical Literature database were systematically searched. Case-control studies on the correlation between serum 25-hydroxyvitamin D levels and gastric cancer were retrieved, and the data extracted were analyzed. The results of 9 case-control studies containing 671 patients showed that serum 25(OH)D levels in the gastric cancer group were lower than those in the control group (weighted mean difference (WMD) = −8.90, 95% confidence interval (CI): −11.5, −6.32, *p* < 0.01); the risk of vitamin D deficiency in the gastric cancer group was higher than that in the control group (Odds ratio = 3.09, 95% CI: 1.96, 4.87, *p* < 0.01). The serum 25(OH)D levels in patients with well and moderately differentiated gastric cancer were higher than those in patients with poorly differentiated gastric cancer (WMD = −3.58, 95% CI: −6.41, −0.74, *p* = 0.01). Thus, low levels of vitamin D may increase the risk of gastric cancer. Systematic review registration: PROSPERO CRD42022327942.

## 1. Introduction

Gastric cancer (GC) is one of the most common malignancies worldwide, with over one million cases being diagnosed yearly [1]. The global incidence and mortality of GC have declined in recent years, but GC remains the third leading cause of cancer-related deaths in China [2]. Meantime, it is estimated to be the fifth most common and third highest mortality cancer in the world and remains an important global clinical and epidemiological problem [3]. Vitamin D (VD) is a hormone that is required for human physiological functions. In addition to playing an important role in calcium and phosphorus metabolism and bone health, VD is also involved in biological processes related to antitumor effects, such as cell proliferation, differentiation, and immune response [4]. Epidemiological studies in China and abroad are also exploring the relationship between VD, 25-hydroxyvitamin D[25(OH)D], which is the detection index in the blood, and GC [5,6,7,8,9]. In a study of factors affecting GC, Ren et al. reported that patients with vitamin D deficiency had higher overall mortality than patients with sufficient vitamin D in GC patients [10]. An experimental study suggested that 1, 25-(OH)D_3_ can induce apoptosis in GC cells, suggesting its use in cancer therapy [11]. A review study by Du et al. suggested that vitamin D may inhibit the viability, proliferation, and metastasis of gastric cancer cells and inhibit Helicobacter-related gastric cancer [12]. Conversely, a meta-analysis study reported no statistically significant relationship between serum vitamin D concentrations and the risk of GC [7]. A prospective study also found that higher serum 25 (OH) D concentrations were significantly associated with ESCC, but not with gastric cancer [13]. The conclusions are not consistent. These associations require further research.

Currently, 25(OH)D is recognized as the best indicator for the objective evaluation of VD status [14,15,16]. Consequently, we used the meta-analysis method to quantitatively analyze studies on VD levels and GC that were systematically retrieved from various databases. We aimed to assess the difference between serum VD levels in patients with GC and the non-cancerous control group, to provide scientific evidence for clinical VD supplementation in the treatment of GC.

## 2. Materials and Methods

### 2.1. Search Strategy and Data Sources

PubMed, Embase, Cochrane Library, Web of Science, The China Academic Journals full-text database, Wanfang Database of Chinese Academic Journals, VIP Chinese Science and Technology Periodicals database, and Chinese Biomedical Literature database were systematically searched. In addition to the above retrieval, we manually traced the references included in the literature. All of the literature on the correlation between VD levels and GC, from the establishment of the databases up to 20 January 2022, were collected. A combination of subject and free words was used for the retrieval process. The following MESH and non-MESH terms were combined in the searches: “Vitamin D”, “Calcitriol”, “Cholecalciferol”, “Ergocalciferol”, “25-Hydroxyvitamin D”, “1, 25-dihydroxyvitamin d”, “25-(0H)-D”, “Stomach Neoplasms”, “Gastric Neoplasms”, “Cancer of Stomach”, “Stomach Cancers”, and “Gastric Cancer”.

### 2.2. Inclusion and Exclusion Criteria of Literature

Inclusion criteria:

(1) Research type: Case-control studies; (2) Research object: Patients (age ≥ 18) with GC who was diagnosed with gastric cancer by gastroscopy and postoperative histopathology, and the control group included healthy people who could be matched with the case group in the same period; (3) Research content: correlation between VD and GC; (4) Outcome indicators: serum 25(OH)D levels; (5) The original data provided the mean, standard deviation, and 95% confidence interval (CI) of the VD levels or they could be calculated.

Exclusion criteria:

(1) Studies with no clear diagnostic criteria for GC; (2) Literature with incomplete or incorrect data that cannot be corrected; (3) Animal experiments, abstracts, reviews, and case reports; (4) Republished literature.

### 2.3. Study Selection and Data Extraction

The literature screening was conducted independently by two investigators (YY Zhou and XH Zou) according to the inclusion and exclusion criteria. Disagreements between the two investigators were resolved by a third investigator (X Liu) through discussions. The titles and abstracts of the literature were read first. When the obvious irrelevant literature was excluded, the full text was read further to determine whether it should be included in the study. The following information was extracted from each study: the last name of the first author, year of publication, country of study, ages of participants, gender, sample size, and methods used for assessing serum 25(OH)D levels. The mean, standard deviation, and range of serum 25(OH)D levels were also extracted; disagreements were resolved as indicated above. If required, we contacted the authors of preliminary studies for more information.

### 2.4. Study Quality Assessment

The risk of bias (RoB) of all of the selected studies was independently assessed by two investigators (ZYY and ZXH), using the Newcastle-Ottawa quality assessment scale [17]. This quality-assessment tool comprises eight items categorized into three groups (selection, comparability, and outcome), with a scoring scale of 0–9 based on selection, comparability, and outcome. The articles that achieved six or more scores were considered to be of high quality.

### 2.5. Statistical Analysis

Review Manager 5.4 and Stata 25.0 software was used for statistical analysis. The continuous outcomes adopted the weighted mean difference (WMD) as the effect indicator, and the dichotomous outcomes adopted the odds ratio (OR) value as the effect indicator. Each effect variable gave the point estimation value and 95% CI. Heterogeneity among studies was assessed using I^2^ statistics; when I^2^ < 50%, it was considered homogeneous, and the fixed-effects model statistical analysis was used. When I^2^ > 50%, it was considered heterogeneous, and the random-effects model statistical analysis was used. Subgroup analysis was used to explore and interpret the sources of heterogeneity, and sensitivity analysis was used to evaluate the stability of the outcome indicators. The funnel plot, Begg’s, and Egger’s tests were employed to assess the publication bias.

## 3. Results

**Figure 1:**
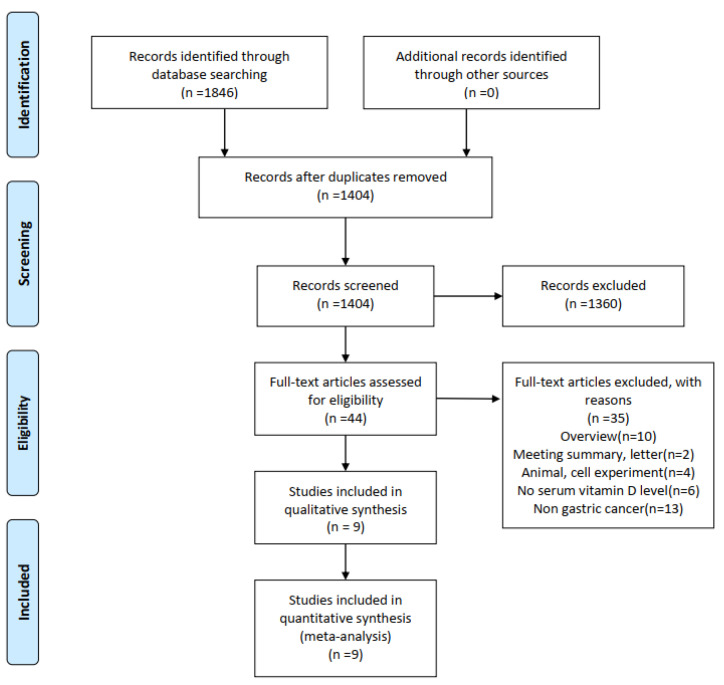
Four-stage flowchart of study selection according to the PRISMA statement.

We conducted a systematic literature search strictly according to the PRISMA statement (Supplementary Materials). A total of 1846 articles were obtained from this initial screening. After excluding duplicates, 1404 articles were scanned by titles and abstracts. Of which, 1360 articles were removed due to obviously irrelevant. The remaining 44 potentially relevant articles were retrieved for full-text assessment. After conditional screening based on the inclusion and exclusion criteria, we selected nine articles for this meta-analysis. The detailed search and study selection process is shown in Figure 1.

### 3.1. Basic Characteristics of the Selected Studies and Results of Bias Risk Assessment

A total of nine case-control studies were finally selected [18,19,20,21,22,23,24,25,26], with a total of 1278 subjects, including 671 patients with GC and 607 healthy controls. The basic characteristics of the selected studies are shown in Table 1, and the results of the risk assessment are shown in Table 2.

### 3.2. Analysis of Serum 25(OH)D Levels in the GC and Control Groups

**Figure 2:**
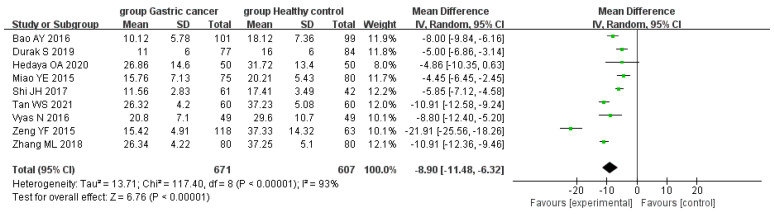
Forest plot of comparison of 25(OH)D levels between the GC and control groups.

Serum 25(OH)D levels in patients with GC and healthy controls were assessed in the nine selected studies [18,19,20,21,22,23,24,25,26]. Testing for overall heterogeneity revealed that there was significant statistical heterogeneity among the selected studies (*p* < 0.01, I^2^ = 93%), and so the random-effects model was used for the analysis. Among the nine selected studies, one study reported that the difference in serum 25(OH)D levels between the GC group and the control group were not significant. The results of the combined effect showed that serum 25(OH)D levels in the GC group were lower than those in the control group, and the difference was significant (WMD = −8.90, 95% CI:−11.48, −6.32, *p* < 0.01) (Figure 2).

### 3.3. Analysis of Serum 25(OH)D Deficiency in the GC and Control Groups

**Figure 3:**
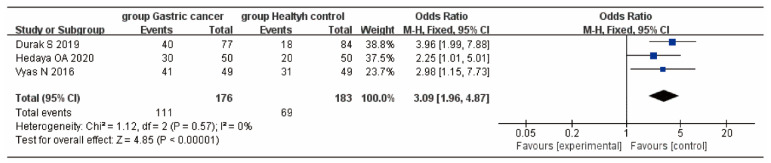
Forest plot of 25(OH)D deficiency comparison between the GC and control groups.

Three studies [19,20,24] described VD insufficiency as 25(OH)D < 30 ng/mL. The heterogeneity test showed that there was no heterogeneity (*p* = 0.57, I^2^ = 0%). The fixed-effects model was used to merge the effect values. The results showed that the risk of VD deficiency in the GC group was higher than that in the control group (OR = 3.09, 95% CI:1.96, 4.87, *p* < 0.01) (Figure 3).

### 3.4. Analysis of Serum 25(OH)D Levels in Patients with GC with Different Cell Differentiation Degrees

**Figure 4:**
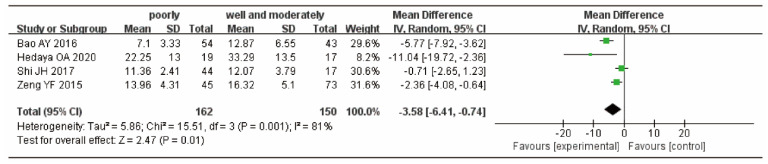
Forest plot of comparison of 25(OH)D in patients with GC with different degrees of cell differentiation.

Four studies [18,20,22,25] provided the serum 25(OH)D levels of patients with GC with different cell differentiation degrees in the GC group, including 162 patients with GC in the low differentiation group and 150 patients with GC in the high and medium differentiation groups. The heterogeneity test results indicated that there was heterogeneity (*p* < 0.01, I^2^ = 81%), and the random-effects model was used to combine the effect values. The results showed that the levels of serum 25(OH)D in patients with high and medium differentiation of GC were higher than those in patients with low differentiation, and the difference was significant (WMD = −3.58, 95% CI: −6.41, −0.74, *p* = 0.01) (Figure 4).

### 3.5. Subgroup Analyses

**Figure 5:**
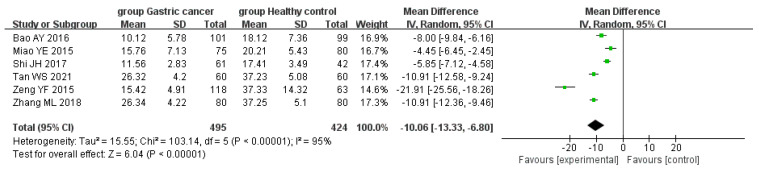
Forest plot of comparison of 25(OH)D levels between the GC and control groups in East Asia.

**Figure 6:**
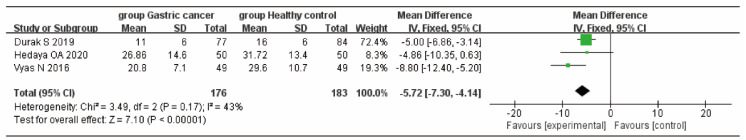
Forest plot of comparison of 25(OH)D levels between the GC and control groups in non-East Asia.

In the meta-analysis comparing serum 25(OH)D levels between the GC and control groups, subgroup analyses were conducted according to the geographical locations of the subjects in East Asia and non-East Asia. Six articles [18,21,22,23,25,26] were authored in East Asia and three articles [19,20,24] in non-East Asia. Eastern Asian subgroup studies showed that serum 25(OH)D levels in patients with GC were lower than those in the control group (WMD = −10.06, 95% CI: −13.33, −6.80, *p* < 0.01), but heterogeneity among the studies was high (*p* < 0.01, I^2^ = 95%) (Figure 5). Subgroup studies in non-East Asia also showed that the serum 25(OH)D levels of patients with GC were lower than those of the control group (WMD = −6.11, 95% CI: −8.65, −3.57, *p* < 0.01), and the heterogeneity test results showed no heterogeneity (*p* < 0.17, I^2^ = 43%) (Figure 6).

### 3.6. Sensitivity Analyses

Meta-analysis was performed again after eliminating individual studies one by one, and the results showed no significant change, suggesting that the stability of the results was good. The meta-analysis comparing serum 25(OH)D levels between the GC and control groups after excluding the study by Zeng et al. [26], showed that heterogeneity decreased (*p* < 0.001, I^2^ = 89%), and there was still a correlation between serum 25(OH)D levels and GC (WMD = −7.49,95% CI: −9.51, −5.46, *p* < 0.01).

### 3.7. Publication Bias

**Figure 7:**
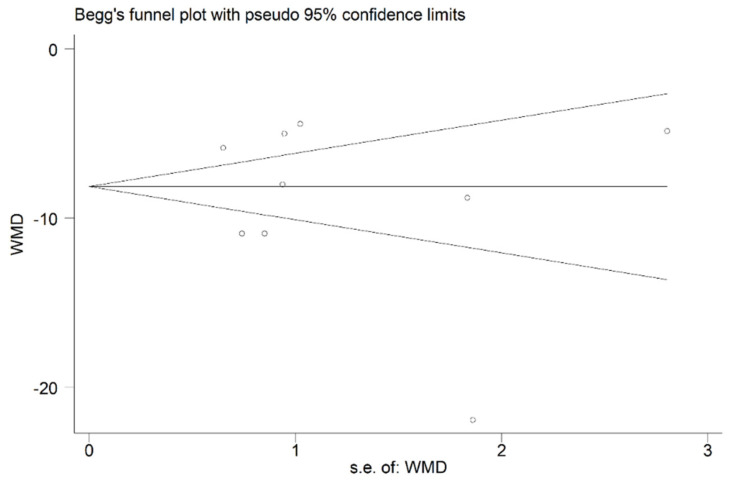
Begg’s test for publication bias testing.

Begg’s and Egger’s tests were used to evaluate the publication bias; Egger’s linear regression test, t = −0.68 (*p* > 0.05) and Begg’s rank correlation test Z = 0.10 (*p* > 0.05). In this meta-analysis, Begg’s and Egger’s tests indicated no publication bias among the selected articles (Figure 7).

## 4. Discussion

VD is a fat-soluble vitamin closely related to human health. The main physiological functions of VD are to regulate calcium, phosphorus, and bone metabolisms, and to maintain blood calcium and phosphorus levels. VD in the human body mainly comes from sunlight irradiation and animal food intake. Currently, the best indicator for evaluating the state and activity of VD in the body is 25(OH)D in circulation. VD deficiency is one of the global health problems; approximately one billion people have VD deficiency and insufficiency [27]. In addition to its physiological functions, VD exerts antitumor effects by regulating the cell cycle, cell differentiation, apoptosis, tumor invasion, and metastasis, and inhibiting angiogenesis. Studies have proven that VD is important for cancer prevention and VD deficiency is associated with a variety of human cancers [28]. Low serum VD levels are closely related to an increased risk of malignant tumors, such as colon, breast, prostate, skin, and cervical cancers, and VD can inhibit tumor occurrence and progression through multiple mechanisms [29,30].

GC is a malignant tumor occurring in the epithelial tissue of the gastric mucosa, and it is the fifth most common cancer and the third leading cause of cancer-related deaths worldwide. In 2020, there were more than one million new GC cases in the world, and 7.69 million deaths [31]. The incidence of GC tends to be high in the younger population [32], which seriously threatens human health and life. The pathogenesis of GC is closely related to many factors, such as heredity, Helicobacter pylori infection, precancerous lesions, environment, and diet [33]. With increasing attention on VD in recent years, it has been found that VD deficiency does not only affect the normal growth of the human body but is also associated with the occurrence and development of cancer. A nested case-control study [34] found that low plasma 25(OH)D concentration (<15.1 ng/mL) was related to an increase in total cancer risk. A prospective study also found low levels of VD may be associated with increased cancer incidence and mortality in men, particularly for digestive-system cancers [6]. The retrospective matched case-control study by Vyas et al. [24], showed that a decrease in serum VD level may increase the risk of gastric adenocarcinoma. Other studies found that low serum 25(OH)D level is a risk factor for GC [5,20]. However, a prospective case-control study [35] from Japan found that although plasma 25(OH)D concentration was negatively correlated with total cancer risk, no significant correlation was found in the GC subgroup. Similarly, the results of a meta-analysis by Khayatzadeh et al. [7]. showed that there was no correlation between serum 25(OH)D levels and GC risk. In addition, a cohort study in the United States [36] found that higher serum 25(OH)D concentration significantly correlated with lower total cancer mortality, but this correlation was not significant in patients with GC. The research results of the correlation between serum 25(OH)D levels and GC risk and mortality are inconsistent.

Nine case-control studies were selected for this study, involving 671 patients with GC. Serum 25(OH)D levels were compared between the GC and control groups. Meta-analysis showed that the levels of VD in the GC group were lower than those in the control group, the risk of VD deficiency in the GC group was higher than that in the control group, and the level of VD was related to the degree of GC cell differentiation. These results suggest that the presence an association between VD level and GC, and a low VD level is likely to increase the risk of GC.

This meta-analysis had high heterogeneity. Further analysis of the age and gender of the two groups showed no statistical difference, suggesting that age and gender may not be the sources of clinical heterogeneity. Since VD level is related to many factors, such as light time, light intensity, geographical location, season, lifestyle, economy, and culture, the VD status of people in different regions differs, which may be the cause of the heterogeneity. Further subgroup analysis showed that the serum VD levels of patients with GC in East Asia and non-East Asia were lower than those in the control group. The heterogeneity of the studies in East Asia was high, but that of the studies in non-East Asia was low. This suggests that the decrease in serum VD levels in patients with GC may be related to racial and dietary differences. In previous studies, it was observed that serum 25(OH)D levels of the participants showed significant differences according to the seasons. This may also be a reason for the high heterogeneity [37,38]. In addition, this study also explored the relationship between serum VD levels and the clinicopathological features of GC. The results showed that the levels of serum 25(OH)D in patients with low differentiation of GC were lower than those in patients with high and medium differentiation. This suggests that the poorer the differentiation, the lower the level of VD in patients with gastric cancer.

There are still some limitations in this study. Only nine pieces of literature were reviewed, and the number of cases was relatively small. In addition, the heterogeneity of the selected studies was high. The factors that affect serum vitamin D levels, such as body mass index (BMI), the season of blood collection, and household income, may also be other reasons for the high heterogeneity [39,40,41,42]. However, due to the limited existing research, the author did not mention these contents in the original study, so no further analysis was made. Moreover, the reviewed literature was retrospective studies; no prospective study confirmed that there may be a certain selection bias. The selected studies are mainly from East Asia, particularly China, and the results are not globally representative.

A prospective KORA F4 study [43] from Germany found no protective effect of 25(OH)D against developing cancer. One limitation of this study is that the overall cancer incidence rate in the cohort was only 37% of the value that would be expected based on the national incidence rates in Germany. Additionally, the overall low levels of serum 25(OH)D among the participants, may not be sufficient to demonstrate the protective effects of 25(OH)D. However, a recently published case-control study [44] from Vietnam showed low dietary intake of VD may associate with an increased risk of GC in the Vietnamese population. This is similar to our Research findings. However, the evidence of epidemiological studies remains paradoxical and inconsistent. There is a strong need for more randomized, controlled trials that would investigate this matter in the future.

## 5. Conclusions

In summary, the results of this meta-analysis showed that the serum VD levels of patients with GC were lower than those of the healthy control group. The risk of VD deficiency in patients with GC was higher than that in the healthy control group, and it was related to the degree of differentiation of cancer cells. The serum VD levels of the high and medium differentiation groups were significantly higher than those of the low differentiation group. In the future, more clinical studies on GC and VD are needed, and it is important to detect the serum VD levels in patients with clinically diagnosed GC, to further study whether VD supplementation can reduce the incidence of GC.

## Figures and Tables

**Table 1:** Characteristics of selected studies.

Author	Year	Area	Sample	Sex (Male/Female)	Age (Years)	Measurement Method of Vitamin D Levels	NOS Grade
			Case-Control	Case-Control			
Bao AY [18]	2016	Wuhan, China	101 99	/ /	≥18	COBAS E60	7
Durak S [19]	2019	Turkey	77 84	33/44 46/38	≥18	HPLC	8
Hedaya OA [20]	2020	Iran	50 50	35/15 27/23	≥18	ELISA	8
Miao YE [21]	2015	Shandong, China	75 80	53/22 57/23	25–75	ELISA	7
Shi JH [22]	2017	Shanxi, China	61 42	45/16 19/23	23–78	ELISA	8
Tan WS [23]	2021	Foshan, China	60 60	35/25 38/22	35–82	ELISA	8
Vyas N [24]	2016	America	49 49	24/25 24/25	≥18	ELISA	8
Zeng YF [24]	2015	Zhengzhou, China	118 63	76/42 /	18–79	ELISA	6
Zhang ML [26]	2018	Yancheng, China	80 80	51/29 50/30	38–80	ELISA	7

**Table 2:** The results of case-control study bias risk assessment (points).

Studies	Selection				Compareability	Exposure			Total Score
	Is the Case Definition	Representativeness of the Cases	Selection of Controls	Definition of Controls		Ascertainment of Exposure	The Same Method of Ascertainment for Cases and Controls	Non-Response Rate	
Bao AY [19]	1	1	1	1	1	1	1	0	7
Durak S [20]	1	1	1	1	2	1	1	0	8
Hedaya OA [21]	1	1	1	1	2	1	1	0	8
Miao YE [22]	1	1	1	1	1	1	1	0	8
Shi JH [23]	1	1	0	1	2	1	1	0	7
Tan WS [24]	1	1	1	1	2	1	1	0	8
Vyas N [25]	1	1	1	1	2	1	1	0	8
Zeng YF [26]	1	1	0	1	1	1	1	0	6
Zhang ML [27]	1	1	1	1	1	1	1	0	7

## Supplementary Materials

The following supporting information can be downloaded at: https://www.mdpi.com/article/10.3390/curroncol29110661/s1. Source of Supplementary Material is Ref. [45].
