# Supplementary material for: Correlation between Serum 25-Hydroxyvitamin D Levels and Gastric Cancer: A Systematic Review and Meta-Analysis
Source: Curr Oncol. 2022 Nov 2;29(11):8390–400. doi: 10.3390/curroncol29110661 (PMC9689382; doi:10.3390/curroncol29110661)
Supplement: Supplementary file 1 [file curroncol-29-00661-s001.zip › curroncol-1966036-Supplemental file S2.pdf]

Supplemental Information file S2

The rationale for conducting the meta-analysis

Gastric carcinoma is the fifth most common cancer worldwide. It is the third most common cause of deaths due to cancer. worldwide, gastric cancer rates are twice as high in men compared to women. American Cancer Society's estimates for stomach cancer in the United States for 2015 revealed that approximately 24590 cases will be diagnosed (15540 in men and 9050 in women) and that 10720 people will die from this type of cancer (6500 men and 4220 women).

Vitamin D has been found to have a role in the tumourigenesis of gastric cancer. However, the correlation between serum vitamin D levels and gastric cancer remains inconsistent. Although the relationship between vitamin D status and risk of gastric cancer was indeterminate and only a possible relationship has been suggested in a few studies it has not been identified whether there is a definitive correlation between vitamin D status and patients with gastric cancer. We aimed to evaluate the serum levels of vitamin D in patients with gastric cancer compared to the non-cancerous control group and provide evidence for clinical vitamin D supplementation in the treatment of gastric cancer.
